# Supplementary material for: Content-rich biological network constructed by mining PubMed abstracts
Source: BMC Bioinformatics. 2004 Oct 8;5:147. doi: 10.1186/1471-2105-5-147 (PMC528731; doi:10.1186/1471-2105-5-147)
Supplement: Additional File 2 — The original results of the above study (non-essential files are deleted to keep the file size under the limit set by BMC bioinformatics). [file 1471-2105-5-147-S2.bz2 › chilibotAdditionalFile2/dip05/480ID8702798E10569/html/IL8RB_CXCL3.html]

 


 **IL8RB** and **CXCL3** 
  
Found 2 abstracts in PubMed, retrieved 2.  
 

 What does Google say? 
 PDF only 
| .edu only 

---

**Interactive relationship** (e.g. stimulation, inhibition, etc)

**Inhibitory relationship**- Substitution into IL 8 of the GRO gamma  [ **CXCL3** ]  sequences corresponding to either the amino terminal loop amino acids 1 18 or the first beta sheet amino acids 18 32 reduced binding to both IL8R1 and IL8R2  [ **IL8RB** ] .  Ref: 8626516 J Biol Chem, 1996
**Neutral relationship**- One such receptor, CXCR2  [ **IL8RB** ] , binds multiple CXC chemokines, including interleukin 8, GRO alpha, GRO beta, GRO gamma  [ **CXCL3** ] , and NAP 2.  Ref: 10747307 Arch Pathol Lab Med, 2000

**Non-interactive relationship** (e.g. studied together, co-existance, homology, etc.)

- When IL 8 sequences were substituted into GRO gamma  [ **CXCL3** ] , a single domain containing the second beta sheet of IL 8 amino acids 18 32 was sufficient to confer high affinity binding for both IL8R1 and IL8R2  [ **IL8RB** ] .  Ref: 8626516 J Biol Chem, 1996
- To determine the regions of interleukin 8 IL 8 that allow high affinity and interleukin 8 receptor type 1 IL8R1 specific binding of chemokines, we produced chimeric proteins containing structural domains from IL 8, which binds to both IL8R1 and interleukin 8 receptor type 2  [ **IL8RB** ]  IL8R2  [ **IL8RB** ]  with high affinity, and from GRO gamma  [ **CXCL3** ] , which does not bind to IL8R1 and binds to IL8R2  [ **IL8RB** ]  with reduced affinity.  Ref: 8626516 J Biol Chem, 1996
